# Platelet-Rich Plasma for COVID-19-Related Olfactory Dysfunction: A Systematic Review and Meta-analysis of Randomized Controlled Trials

**DOI:** 10.7759/cureus.94386

**Published:** 2025-10-12

**Authors:** Ebraheem Albazee, Saad A Alajmi, Ali M Alkandari, Abdullah N Aladwani, Yaqoub Y Alenezi, Munawer A Alsaeed, Bader Uqlah, Ahmed Abu-Zaid

**Affiliations:** 1 Otorhinolaryngology-Head and Neck Surgery, Kuwait Institute for Medical Specializations (KIMS), Kuwait City, KWT; 2 Otolaryngology-Head and Neck Surgery, Al-Jahra Hospital, Al-Jahra, KWT; 3 Medicine and Surgery, Kuwait Institute for Medical Specializations (KIMS), Kuwait City, KWT; 4 Medicine and Surgery, College of Medicine, Arabian Gulf University, Manama, BHR; 5 Biochemistry and Molecular Medicine, College of Medicine, Alfaisal University, Riyadh, SAU

**Keywords:** anosmia, olfaction, otolaryngology, platelet-rich plasma, prp

## Abstract

A notable rise in olfactory dysfunction (OD) prevalence has been observed since the COVID-19 pandemic. COVID-19-related OD is associated with several consequences, especially deteriorated quality of life. Hence, several treatment options have been investigated, with platelet-rich plasma (PRP) showing promising results. A systematic review and meta-analysis summarizing randomized controlled trial (RCT) evidence were retrieved from PubMed, Google Scholar, Scopus, and Web of Science up to June 2025. The risk of bias was assessed using the Cochrane Risk of Bias 2 assessment tool. Data were analyzed using Stata MP version 18 (StataCorp LLC, College Station, TX), pooling dichotomous outcomes as relative risks (RRs) and continuous outcomes as standardized mean differences (SMDs), each with 95% confidence intervals (CIs). Four RCTs, including 198 participants, were included in our meta-analysis. PRP significantly improved objective olfactory scores (SMD = 1.86, 95% CI (0.14, 3.57), p = 0.03) and subjective olfactory scores (SMD = 0.92, 95% CI (0.32, 1.51), p < 0.001). Additionally, PRP significantly increased the response rate (RR = 1.79, 95% CI (1.14, 2.81), p = 0.01). PRP was generally well-tolerated across the included trials, with no major adverse events reported. Two RCTs showed an overall low risk of bias, one trial showed some concerns, and another showed a high risk of bias. With uncertain evidence, PRP may improve both objective and subjective smell function and clinical outcomes in people with long COVID-related OD. PRP treatment was reported to be safe, with minor, temporary side effects primarily related to the procedure. Although initial results are promising, the small number of RCTs requires a cautious approach to interpretation.

## Introduction and background

Since the COVID-19 pandemic, there has been a considerable increase in the prevalence of olfactory dysfunction (OD) [1,2]. OD encompasses a range of disorders, including anosmia (complete loss of smell), hyposmia (reduced ability to smell), and parosmia (distorted perception of smells). Studies show that 26.5% to 46% of people experience COVID-related OD one year after infection, dropping to 8.3% after two years [3]. Recent research shows partial smell recovery in up to 54.3% and complete recovery in 38.2% of patients within two years of infection [4,5]. Yet, subjective reports of smell function in anosmic patients do not strongly correlate with psychophysical measures [6]. While approximately 79% of participants in a recent study reported subjective recovery 36 weeks post-COVID-19 infection, a significantly smaller percentage (51.5%) achieved normal Brief Smell Identification Test (BSIT) scores [7]. Objective psychophysical testing can reveal dysfunction even in patients who do not report smell difficulties [8]. Patients with OD experience various negative consequences, such as altered taste, weight fluctuations, social withdrawal, poor hygiene, reduced quality of life, and a higher mortality rate [9]. Smell training has shown limited but consistent success across various causes of OD and is commonly prescribed [10]. Nonetheless, additional treatment options remain warranted for optimal management.

Platelet-rich plasma (PRP), an autologous concentrate of platelets rich in growth factors, presents a promising approach for managing OD [11]. Platelet activation triggers the release of a complex mixture of bioactive molecules, such as platelet-derived growth factor, transforming growth factor beta, vascular endothelial growth factor, and nerve growth factor, collectively promoting tissue repair, angiogenesis, cellular proliferation, and neurogenesis [12-14]. Due to the inherent regenerative capacity of the olfactory neuroepithelium, it is posited that PRP enhances this natural process by supporting the survival of olfactory sensory neurons, promoting neurite outgrowth, and modulating local inflammation [15], with its autologous nature ensuring a favorable safety profile. Given its multimodal approach to inflammation, degeneration, and impaired regeneration and its proven success in other regenerative medicine areas, PRP presents a compelling rationale for its application in olfactory system regeneration [14].

Emerging clinical evidence from randomized controlled trials (RCTs) suggests that PRP may be beneficial for COVID-19-related OD [6,11,16,17]. Given the significant burden of persistent post-COVID-19 OD, the limitations of current treatment options, and the promising yet heterogeneous emerging evidence for PRP, a comprehensive systematic review and meta-analysis are warranted. Therefore, we performed this systematic review and meta-analysis to investigate the efficacy and safety of PRP for the management of COVID-related OD.

## Review

Methodology

This systematic review and meta-analysis were conducted in accordance with the Preferred Reporting Items for Systematic reviews and Meta-Analyses (PRISMA) statement [18] and the Cochrane Handbook for Systematic Reviews of Interventions [19].

Data Sources and Search Strategy

On June 05, 2025, an electronic search was conducted on the following databases: Web of Science (WOS), PubMed, Scopus, and Google Scholar. The search strategy included the following search entries: “(COVID-19 OR SARS-CoV-2 OR "Coronavirus disease 2019" OR "Post-COVID-19 Syndrome" OR "Long COVID" OR "Post COVID" OR "Persistent COVID") AND ("Platelet-Rich Plasma" OR PRP) AND ("Olfactory Dysfunction" OR "Smell Disorder" OR "Smell Dysfunction" OR Anosmia OR Hyposmia OR Parosmia OR Phantosmia OR Olfaction OR "Smell Loss" OR "Olfactory Loss")”. Our search strategy employed unrestricted access to most databases; however, Scopus searches were limited to the title. Table 1 provides detailed information on the specific search terms and results for each database. To maximize the completeness of our search, we conducted a manual screening of the reference lists of all included studies and examined key clinical trial registries such as ClinicalTrials.gov and the WHO International Clinical Trials Registry Platform (ICTRP). Furthermore, supplementary sources, including ResearchGate, were reviewed to capture any relevant unpublished or ongoing studies. No restrictions were applied on the basis of language, country of origin, or publication status.

**Table 1:** Search strategy

Database	Search terms	Search field	Search results
PubMed	(COVID-19 OR SARS-CoV-2 OR "Coronavirus disease 2019" OR "Post-COVID-19 Syndrome" OR "Long COVID" OR "Post COVID" OR "Persistent COVID") AND ("Platelet-Rich Plasma" OR PRP) AND ("Olfactory Dysfunction" OR "Smell Disorder" OR "Smell Dysfunction" OR Anosmia OR Hyposmia OR Parosmia OR Phantosmia OR Olfaction OR "Smell Loss" OR "Olfactory Loss")	All fields	20
Web of Science	(COVID-19 OR SARS-CoV-2 OR "Coronavirus disease 2019" OR "Post-COVID-19 Syndrome" OR "Long COVID" OR "Post COVID" OR "Persistent COVID") AND ("Platelet-Rich Plasma" OR PRP) AND ("Olfactory Dysfunction" OR "Smell Disorder" OR "Smell Dysfunction" OR Anosmia OR Hyposmia OR Parosmia OR Phantosmia OR Olfaction OR "Smell Loss" OR "Olfactory Loss")	All fields	22
SCOPUS	TITLE-ABS-KEY ( ( covid-19 OR sars-cov-2 OR "Coronavirus disease 2019" OR "Post-COVID-19 Syndrome" OR "Long COVID" OR "Post COVID" OR "Persistent COVID" ) AND ( "Platelet-Rich Plasma" OR prp ) AND ( "Olfactory Dysfunction" OR "Smell Disorder" OR "Smell Dysfunction" OR anosmia OR hyposmia OR parosmia OR phantosmia OR olfaction OR "Smell Loss" OR "Olfactory Loss" ) )	Title, abstract, and keywords	24
Google Scholar	(COVID-19 OR SARS-CoV-2 OR "Coronavirus disease 2019" OR "Post-COVID-19 Syndrome" OR "Long COVID" OR "Post COVID" OR "Persistent COVID") AND ("Platelet-Rich Plasma" OR PRP) AND ("Olfactory Dysfunction" OR "Smell Disorder" OR "Smell Dysfunction" OR Anosmia OR Hyposmia OR Parosmia OR Phantosmia OR Olfaction OR "Smell Loss" OR "Olfactory Loss")	All fields	99

Eligibility Criteria

RCTs conducted using the following PICO criteria were included: population (P), patients with post-COVID-19 OD; intervention (I), application of PRP; control (C), standard care or saline placebo; and outcomes (O): the primary outcome was the change in objective olfactory scores, including the Sniffin’ Sticks test (threshold, discrimination, and identification (TDI) score), Connecticut Chemosensory Clinical Research Center (CCCRC) olfaction test, and BSIT (for all of these instruments, a higher score indicates a better sense of smell). Secondary outcomes included the change in subjective olfactory scores, including visual analog scale (VAS) and SCENTinel intensity rating; response rate, defined as a significant improvement in symptoms; and adverse events. Furthermore, our analysis excluded quasi-experimental trials, conference presentations and proceedings, observational studies, in vitro research, and review articles.

The Process of Study Selection

Screening was performed in Covidence by two independent reviewers (EA and AA). After duplicate removal, titles and abstracts were initially screened, and then the full texts of potentially eligible studies were reviewed. Disagreements were addressed through discussion until consensus was achieved.

Data Extraction

A preliminary extraction of eligible publications was performed to develop an Excel (Microsoft Corp., Redmond, WA) extraction form. The form was structured into three sections: (1) summary characteristics of the included trials (study ID, country, study design, sample size, treatment protocols, main inclusion criteria, used scores, response definition, primary outcome, and follow-up duration); (2) baseline characteristics of the included participants (age, gender, symptoms duration, and baseline olfactory scores); and (3) the outcomes sheet (change in objective olfactory score, change in subjective olfactory score, response rate, and adverse events).

Two reviewers (EA and AA) independently extracted the data, resolving disagreements through discussion with a senior author. Dichotomous data were documented as event rates, whereas continuous data were summarized using means and standard deviations. Mean and standard deviation were calculated from the median and interquartile range (or range) reported in some included studies using the conversion formulas presented by Wan et al. [20].

Risk of Bias and Certainty of Evidence

The risk of bias of the included studies was assessed using the revised Cochrane Collaboration tool for RCTs (ROB 2) [21]. Two independent reviewers (EA and AA) evaluated each study across key domains, including selection, performance, reporting, and attrition biases, as well as other potential sources of bias. Any disagreements were resolved through consensus. In addition, the certainty of the evidence was appraised using the Grading of Recommendations, Assessment, Development, and Evaluation (GRADE) framework [22,23]. This assessment considered risk of bias, inconsistency, imprecision, indirectness, and potential publication bias. Each judgment was carefully documented and justified, and any differences between reviewers were settled by discussion.

Meta-Analysis

Data were analyzed using Stata MP version 18 (StataCorp LLC, College Station, TX). Dichotomous outcomes were pooled as risk ratios (RRs), and continuous outcomes as mean differences, each with 95% confidence intervals (CIs). Because olfactory scores varied across the included trials, the standardized mean difference (SMD) was applied for pain-related outcomes. A fixed-effect model was the primary analytical approach; a random-effect model was used when significant heterogeneity was detected. Heterogeneity was evaluated using the chi-squared test and the I² statistic, with p < 0.1 for the chi-square test or I² ≥ 50% indicating substantial heterogeneity. Publication bias was not assessed because all evaluated outcomes included fewer than 10 RCTs [24].

Results

Search Results and Study Selection

**Figure 1:**
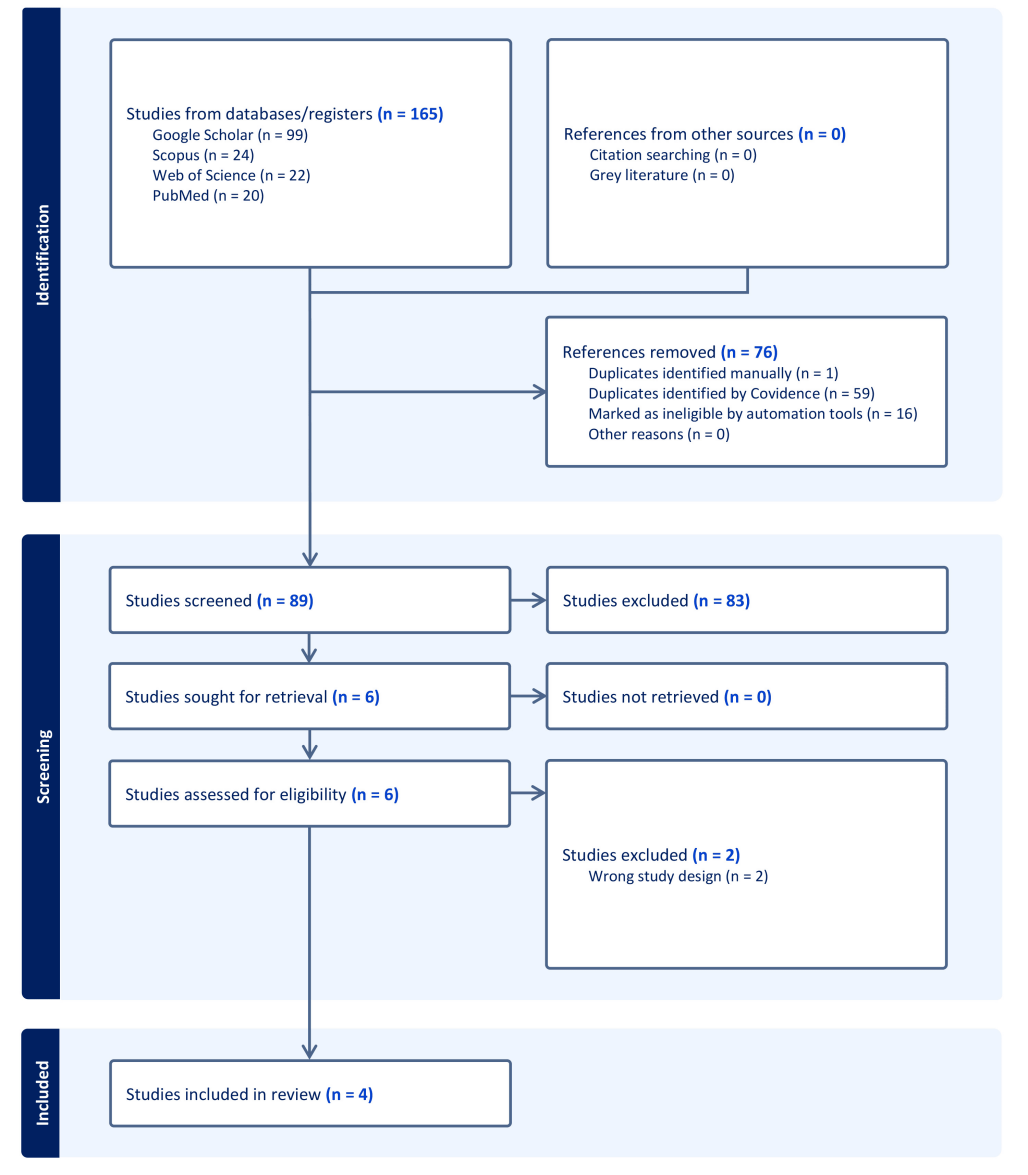
The Preferred Reporting Items for Systematic reviews and Meta-Analyses (PRISMA) flow chart of the screening process

A total of 165 records were identified through the database search. Covidence automatically removed 59 duplicate records, leaving 89 for screening. After title and abstract screening, 83 studies did not meet the inclusion criteria and were excluded. Six full-text articles were assessed for eligibility, of which two were excluded. Ultimately, four RCTs [6,11,16,17] were left to be included in qualitative and quantitative analyses (Figure 1).

Characteristics of Included Studies

Four trials and 198 participants were included in our analysis [6,11,16,17]. Two trials were conducted in the US [6,11], one in Egypt [17], and another in Turkey [16]. The follow-up duration ranged from one to 12 months. Intervention details and the assessed scores are clarified in Table 2. Further baseline data of the included patients are in Table 3.

**Table 2:** Summary characteristics of the included randomized controlled trials BSIT, Brief smell identification test; CCCRC, Connecticut Chemosensory Clinical Research Center; NA, Not available; PRP, Platelet-rich plasma; RCT, Randomized controlled trial; TDI, Threshold, discrimination, identification; VAS, Visual analog scale

Study ID	Study design	Country	N	PRP	Control	Main inclusion criteria	Objective olfactory score	Subjective olfactory score	Response definition	Primary outcome	Follow-up
												Abo El Naga et al. 2022 [17]	RCT	Egypt	60	Three PRP injections in the olfactory cleft at three-week intervals	Olfactory training, topical corticosteroids, omega-3, vitamin B12, and zinc for six weeks	Age >18 years with post-COVID parosmia, unresponsive to 3 months of conservative treatment	NA	NA	NA	VAS for parosmia	1 month
Duffy et al. 2024 [6]	RCT	USA	83	3 monthly treatments of PRP-coated Surgifoam in bilateral olfactory clefts	Monthly treatments of saline-coated Surgifoam	Age ≥18 years, post-COVID olfactory dysfunction ≥6 months, BSIT score ≤8/12, or SCENTinel intensity ≤40/100	BSIT	SCENTinel intensity rating	Change in BSIT ≥ 1	Change in BSIT score	12 months
Evman and Cetin 2023 [16]	RCT	Turkey	25	A single 1 mL PRP injection into the olfactory cleft	No treatment	Age >18 years, post-COVID olfactory dysfunction >1 year, unresponsive to 1 month of conventional treatment	CCCRC olfaction test	NA	NA	CCCRC test scores	1 month
Yan et al. 2022 [11]	RCT	USA	30	Three submucosal PRP injections (1 mL) in bilateral olfactory clefts at two-week intervals	Three submucosal sterile saline injections	Age >18 years, post-COVID smell loss for 6-12 months, UPSIT score ≤33, failed olfactory training and budesonide irrigations	Sniffin' Sticks (TDI) score	VAS	≥5.5 points on the Sniffin' Sticks total score	Change in Sniffin' Sticks (TDI) score	3 months

**Table 3:** Baseline characteristics of the participants BSIT, Brief smell identification test; NA, Not available; OD, Olfactory dysfunction; PRP, Platelet-rich plasma; SD, Standard deviation; SIC, Smell intensity category; STC, Smell threshold category; TDI, Threshold, discrimination, identification; VAS, Visual analog scale

Study ID	Number of patients in each group		Age (years), mean (SD)		Gender (female), %		Mean duration of OD (months), mean (SD)		Baseline olfactory test, mean (SD)	
	PRP	Control	PRP	Control	PRP	Control	PRP	Control	PRP	Control
											Abo El Naga et al. 2022 [17]	30	30	28.9 (6.31)	30.07 (5.74)	63.3	70	NA	NA	VAS: 9.13 (0.73)	VAS: 9.27 (0.78)
Duffy et al. 2024 [6]	41	42	50 (15)	51 (15)	66	76	18 (7)	19 (6)	BSIT: 5.32 (2.35), SCENTinel: 36 (27)	BSIT: 6.10 (2.25), SCENTinel: 39 (29)
Evman and Cetin 2023 [16]	12	13	31.8 (6.9)	33.5 (11.1)	50	53.8	NA	NA	STC: 5.63 (0.68), SIC: 11.42 (1.17)	STC: 5.69 (0.66), SIC: 11.20 (1.12)
Yan et al. 2022 [11]	18	12	44.6 (12.7)	43.4 (16.3)	50	50	8.9 (2.2)	8.6 (2.4)	Sniffin' Sticks (TDI): 24.3 (6.4)	Sniffin' Sticks (TDI): 26.0 (4.4)

Risk of Bias and Certainty of Evidence

**Figure 2:**
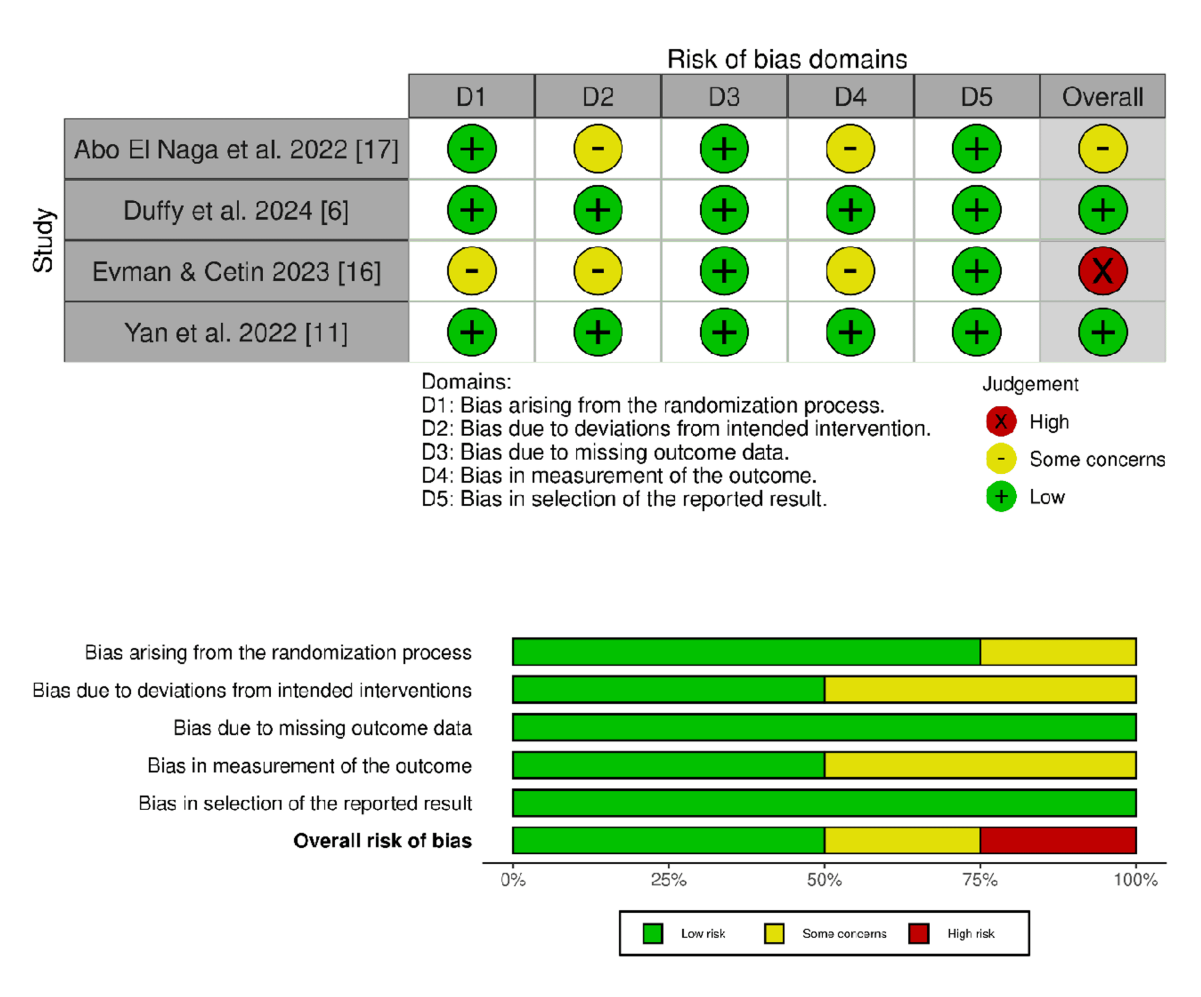
Summary and graphical presentation of risk of bias assessments for all included randomized controlled trials [6,11,16,17]

Two RCTs showed an overall low risk of bias [6,11]. Abo El Naga et al. raised some concerns regarding open lapel interventions and open lapel assessment of subjective outcomes [17]. Evman and Cetin showed a high risk of bias due to the lack of data on the randomization process, open-label interventions, and open-label assessment of subjective outcomes (Figure 2) [16].

Certainty of evidence assessment is demonstrated in Table 4.

**Table 4:** GRADE evidence profile CI, Confidence interval; GRADE, Grading of Recommendations, Assessment, Development, and Evaluation; RCT, Randomized controlled trial; RR, Risk ratio; SD, Standard deviation; SMD, Standardized mean difference a. Evman and Cetin showed a high risk of bias, with >30% of the pooled analysis weight. b. I2 > 75%. c. A wide confidence interval, with a low number of patients. d. A wide confidence interval, with a low number of events.

Certainty assessment							Summary of findings				
Participants	Risk of bias	Inconsistency	Indirectness	Imprecision	Publication bias	Overall certainty of evidence	Study event rates (%)		Relative effect	Anticipated absolute effects	
(studies)									(95% CI)		
Follow-up							With control	With PRP		Risk with control	Risk difference with PRP
Objective olfactory score											
198	Serious^a^	Very serious^b^	Not serious	Very serious^c,d^	None	⨁◯◯◯	-	-	-	-	SMD 1.86 SD higher
(3 RCTs)						Very low^a,b,c,d^					0.14 higher to 3.57 higher
Subjective olfactory score											
113	Not serious	Not serious	Not serious	Very serious^c^	None	⨁⨁◯◯	-	-	-	-	SMD 0.92 SD higher
(2 RCTs)						Low^c^					0.32 higher to 1.51 higher
Response rate (symptoms improvement)											
169	Not serious	Not serious	Not serious	Very serious^d^	None	⨁⨁◯◯	39/84 (46.4%)	69/85 (81.2%)	RR: 1.79	39/84 (46.4%)	367 more per 1,000
(3 RCTs)						Low^d^			1.14 to 2.81		From 65 more to 840 more

Change in Objective Olfactory Score

**Figure 3:**
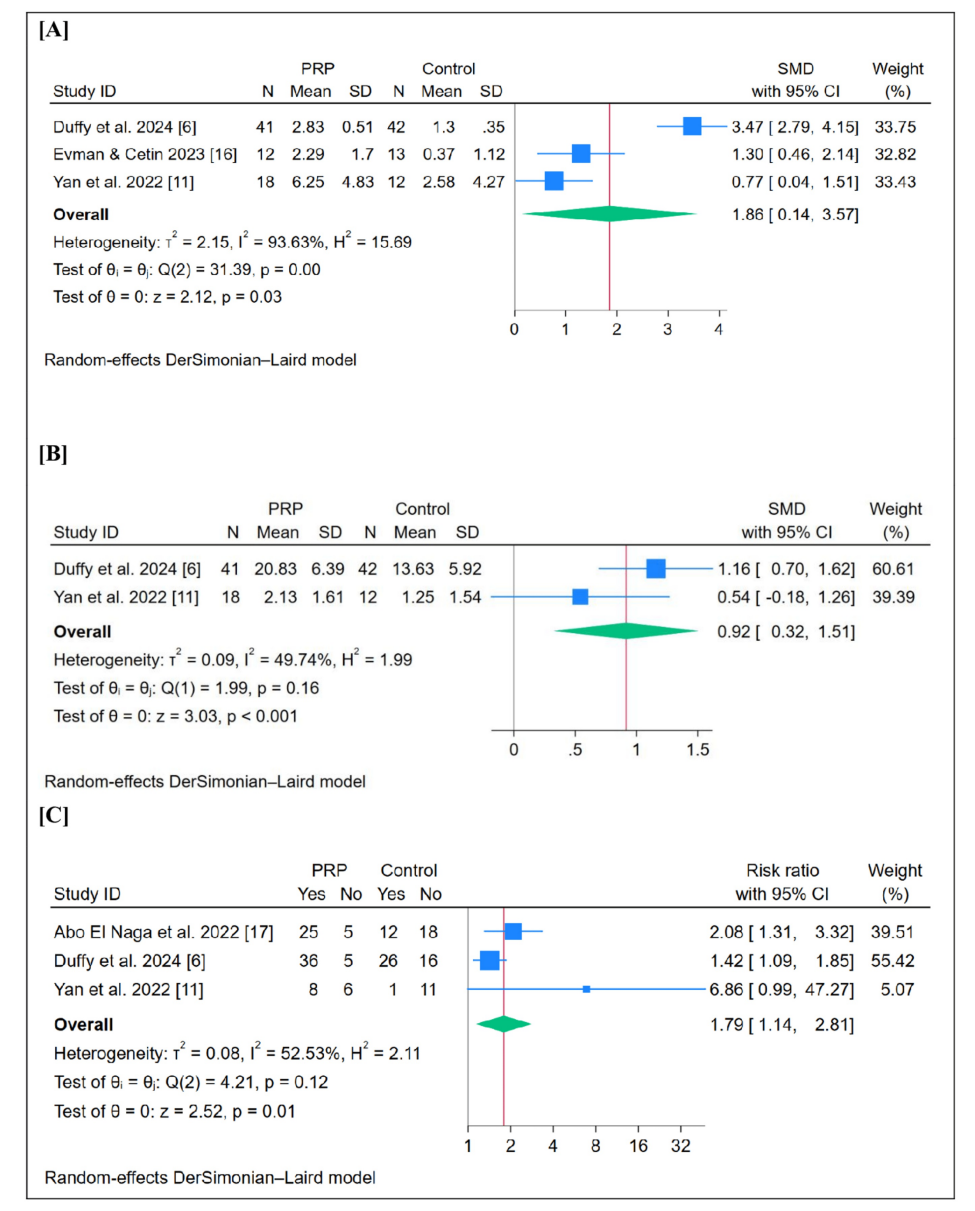
Forest plots of the assessed outcomes: [A] objective olfactory score, [B] subjective olfactory score, and [C] response (symptom improvement) [6,11,16,17] CI, Confidence interval; PRP, Platelet-rich plasma; SD, Standard deviation; SMD, Standardized mean difference

PRP significantly improved objective olfactory scores (SMD = 1.86, 95% CI (0.14, 3.57), p = 0.03) (Figure 3A). Pooled studies were heterogeneous (I^2^ = 93.63%).

Change in Subjective Olfactory Score

PRP significantly improved subjective olfactory scores (SMD = 0.92, 95% CI (0.32, 1.51), p < 0.001) (Figure 3B). Pooled studies were heterogeneous (I^2^ = 49.74%).

Response Rate

PRP significantly increased the response rate (RR = 1.79, 95% CI (1.14, 2.81), p = 0.01) (Figure 3C). Pooled studies were heterogeneous (I^2^ = 52.53%).

Change in Objective Olfactory Score (Sensitivity Analysis)

**Figure 4:**
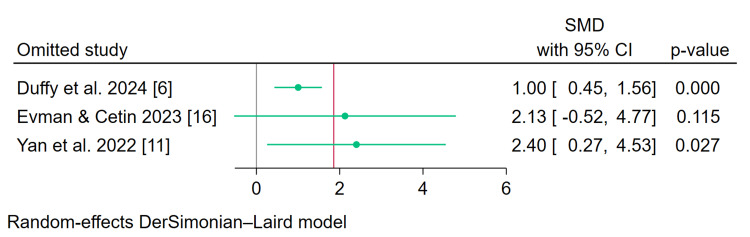
Leave-one-out sensitivity analysis of the change in objective olfactory score [6,11,16] CI, Confidence interval; SMD, Standardized mean difference

Leave-one-out sensitivity analysis showed consistent results, except after the exclusion of Evman and Cetin, which revealed no difference between the two groups (p = 0.115) (Figure 4).

Adverse Events and Tolerability

PRP was generally well-tolerated across the included trials [6,11,16,17]. Abo El Naga and Evman and Cetin reported no adverse events [16,17]. Yan et al. noted minor, short-term side effects, including nasal congestion and pressure, in both the PRP and placebo arms, with one placebo participant experiencing transient photophobia [11]. Similarly, Duffy et al. reported transient sequelae, including pressure, congestion, or headache, following the procedure [6]. Regarding tolerability, two patients in the placebo group withdrew due to anxiety and frustration with the testing methods [6].

Discussion

Summary of the Review and Main Findings

The COVID-19 pandemic has underscored the high incidence of OD, with a substantial number of patients presenting with anosmia as a primary symptom, frequently accompanied by gustatory dysfunction [25]. The significant predictive value of anosmia for COVID-19, particularly in asymptomatic individuals, underscores the urgent need for enhanced therapeutic approaches [26, 27]. Despite the high prevalence and substantial effects of OD, long-term therapeutic approaches remain inadequate [28]. This is primarily due to a lack of robust empirical evidence resulting from underfunded research, insufficient sample sizes, and methodological heterogeneity, which limit the generalizability of the findings [29]. However, the global impact of the COVID-19 pandemic has prompted this field, leading to a notable increase in publications and registered clinical trials focused on treating post-viral OD, including the recent studies on PRP [30].

The pooled analysis of four RCTs involving 198 participants demonstrated that PRP led to statistically significant improvements across all assessed efficacy outcomes compared with control. PRP was associated with a significant enhancement in both objective and subjective olfactory scores, as well as an increased rate of symptom improvement. The consistent results across clinician assessments, device measurements, and patient reports suggest that PRP may be a useful treatment for COVID-related OD. However, these results must be interpreted cautiously, as the GRADE assessment indicates very low to low certainty of evidence.

The observed benefits of PRP are biologically plausible. The pathophysiology of COVID-associated OD is hypothesized to involve inflammation and damage to the olfactory neuroepithelium, predominantly affecting sustentacular cells, rather than direct neuronal infection in many instances [31]. PRP is rich in several growth factors, including platelet-derived growth factor, transforming growth factor-beta, vascular endothelial growth factor, and nerve growth factor [14], which are reported to promote tissue repair, angiogenesis, cellular proliferation, and neurogenesis [32]. The regenerative potential of the olfactory neuroepithelium, facilitated by globose and horizontal basal stem cells [33], may be augmented by PRP, thereby improving olfactory sensory neuron survival, neurite outgrowth, and the modulation of local inflammation [32]. Therefore, the results of this meta-analysis are consistent with an accumulating body of evidence suggesting that PRP has beneficial effects for OD of varied etiologies [14]. Additionally, although the precise mechanisms remain unclear, studies indicate that PRP promotes neurogenesis and regeneration of olfactory neurons and supporting cells, concurrently restoring the integrity of the nasal mucosa [34].

Across the included RCTs, PRP was generally well-tolerated. Abo El Naga et al. and Evman and Cetin reported no adverse events [16,17]. Yan et al. noted minor, short-term side effects, such as nasal congestion and pressure, in both PRP and placebo groups, with one placebo participant experiencing transient photophobia [11]. Similarly, Duffy et al. reported transient sequelae, including pressure, congestion, or headache, following the procedure in both arms [6]. None of the trials reported any serious adverse events attributable to PRP [6,11,16,17]. This favorable safety profile aligns with the established use of PRP in various medical and otolaryngological applications, including androgenetic alopecia [35], tympanic membrane perforation repair [36], and vocal fold regeneration [37], where its autologous nature ensures minimal risk of immunogenicity or disease transmission. The favorable safety profile represents a considerable advantage, especially if efficacy can be conclusively demonstrated for a condition causing significant patient distress.

AlRajhi et al. [38] conducted a systematic review of four studies (two RCTs and two observational studies) involving 233 patients, predominantly female, experiencing anosmia, hyposmia, or parosmia related to COVID-19. The review aimed to assess the efficacy and safety of PRP injections for COVID-19-related OD. While PRP showed potential subjective improvement, particularly in TDI scores and other olfactory assessments, the evidence was insufficient to draw definitive, statistically significant conclusions due to study heterogeneity and limited sample sizes. In contrast, Albazee et al. [39] performed a systematic review and meta-analysis of six RCTs to evaluate PRP use following endoscopic sinus surgery in patients with chronic rhinosinusitis. PRP was associated with improved wound healing, reduced inflammation, and enhanced overall surgical outcomes. However, no statistically significant differences were observed between the PRP and control groups regarding anosmia duration or olfactory function scores, despite a trend toward higher scores in the PRP group. Our findings align with a recent meta-analysis by Bae et al. [40], which also investigated PRP for persistent post-COVID-19 OD. Their analysis of five studies, including observational studies and 254 participants, found that PRP led to a significant improvement in olfactory scores when compared to control groups (SMD: 1.44; 95% CI (0.59, 2.28)) and substantially increased the odds of recovery (odds ratio: 8.66; 95% CI (2.98, 25.23)) [40]. These results are consistent with our findings, which also demonstrated a significant benefit with PRP. Notably, both our meta-analysis and the one by Bae et al. [40] identified a high degree of heterogeneity, underscoring that variations in PRP protocols, patient populations, and outcome assessments remain a significant challenge in the current body of evidence.

Strengths and Limitations

To the best of our knowledge, as of June 2025, this is the first RCT-based meta-analysis to synthesize evidence on the efficacy of PRP for COVID-19-related OD. Additionally, we have strictly adhered to the Cochrane guidelines during the conduct of this meta-analysis and the PRISMA guidelines during its reporting. Still, our analysis is limited by the following: first, the small number of eligible RCTs (four studies) and the limited number of participants (198 patients), which inherently limits the statistical power of the meta-analysis and the precision of the pooled effect estimates. Second, the lack of blinding in some studies, where interventions or outcome assessments are not concealed, is problematic due to the potential for placebo effects in procedural interventions. Third, some key differences in the PRP protocols, including preparation, route of administration, and number of sessions, can lead to the noted heterogeneity. Fourth, different psychophysical tests (BSIT, CCCRC, and Sniffin' Sticks) and subjective scales (VAS and SCENTinel) were used, each with varying properties and sensitivities. Still, we used SMD, as advised, to pool data from these scores. Fifth, the follow-up periods ranged from one to 12 months, which may lead to some heterogeneity in the pooled analysis. Sixth, we did not prospectively register our review protocol, while this is the best practice for systematic reviews, the PRISMA guidelines consider it an optional step in the systematic review process.

Finally, a critical consideration in interpreting these results is the natural history of post-COVID-19 OD, which can include spontaneous recovery, even after several months [41]. The included trials mitigated the influence of spontaneous recovery by enrolling only patients with long-standing OD. Specifically, the baseline requirement was symptoms persisting for six months or longer (Duffy et al. [6] and Yan et al. [11]) or for over one year (Evman and Cetin [16]). Notably, certain studies demonstrated improvements even in control groups, thus underscoring the necessity of rigorous study designs. Also, the procedural aspects of PRP administration (injections or topical application) are likely to generate a substantial placebo effect [42]. This highlights the critical need for rigorous blinding and suitable control groups in subsequent trials; this shortcoming contributed to the "some concerns" or "high" risk of bias assessments in studies with open-label elements.

Implications for Future Research

Future research should focus on several key areas to overcome current limitations and provide stronger evidence of PRP's utility for COVID-19-related OD. First, there is a critical need for large-scale, multicenter, high-quality RCTs to draw more definitive conclusions. Second, standardizing PRP protocols is crucial for making future trials more comparable and interpretable. Standardized techniques are necessary for PRP administration, including the distinction between injection and topical application, precise anatomical targeting, accurate volume measurement, and appropriate frequency. Third, the adoption of core outcome sets (COS) for clinical trials in post-viral OD is urgently needed [43]. Using COS ensures consistent measurement and reporting of key outcomes across studies, making future meta-analyses more feasible. Fourth, future trials should incorporate extended follow-up periods, ideally at least 12 to 24 months, to assess the durability of any observed treatment effects and monitor for potential late-onset adverse events. Fifth, head-to-head comparisons are needed to optimize treatment strategies by evaluating different PRP formulations (e.g., leukocyte-rich versus leukocyte-poor), concentrations, and administration methods. Finally, since olfactory training is a recommended treatment for post-viral OD, future trials should investigate PRP as an adjunctive therapy. These studies could compare combined therapy (PRP plus olfactory training) against appropriate comparators, such as olfactory training alone or olfactory training with a sham PRP procedure.

## Conclusions

PRP may offer therapeutic benefits for improving objective and subjective olfactory function, as well as clinical response rates, in individuals with persistent COVID-related OD. PRP has been reported to be safe, with minor and transient adverse events primarily related to the procedure itself. While the preliminary findings are encouraging, the limited number of RCTs included warrants cautious interpretation of the results.
